# SARS-CoV-2/COVID-19: a primer for cardiologists

**DOI:** 10.1007/s12471-020-01475-1

**Published:** 2020-07-15

**Authors:** A. A. F. de Vries

**Affiliations:** Laboratory of Experimental Cardiology, Department of Cardiology, Leiden University Medical Centre, Leiden, The Netherlands

**Keywords:** Coronavirus disease 2019, Severe acute respiratory syndrome coronavirus 2, Pandemic, Acute respiratory distress syndrome, Vaccine, Antiviral drug

## Abstract

In the late autumn of 2019, a new potentially lethal human coronavirus designated severe acute respiratory syndrome coronavirus 2 (SARS-CoV-2) emerged in Wuhan, China. The pandemic spread of this zoonotic virus has created a global health emergency and an unprecedented socioeconomic crisis. The severity of coronavirus disease 2019 (COVID-19), the illness caused by SARS-CoV‑2, is highly variable. Most patients (~85%) develop no or mild symptoms, while others become seriously ill, some succumbing to disease-related complications. In this review, the SARS-CoV‑2 life cycle, its transmission and the clinical and immunological features of COVID-19 are described. In addition, an overview is presented of the virological assays for detecting ongoing SARS-CoV‑2 infections and the serological tests for SARS-CoV-2-specific antibody detection. Also discussed are the different approaches to developing a COVID-19 vaccine and the perspectives of treating COVID-19 with antiviral drugs, immunomodulatory agents and anticoagulants/antithrombotics. Finally, the cardiovascular manifestations of COVID-19 are briefly touched upon. While there is still much to learn about SARS-CoV‑2, the tremendous recent advances in biomedical technology and knowledge and the huge amount of research into COVID-19 raise the hope that a remedy for this disease will soon be found. COVID-19 will nonetheless have a lasting impact on human society.

**Electronic supplementary material:**

The online version of this article (10.1007/s12471-020-01475-1) contains supplementary material, which is available to authorized users.

## Introduction

Coronaviruses (CoVs) were first described in the 1930s and first visualised (by electron microscopy) in the 1960s [1, 2]. CoVs own their name to the pedal-shaped protrusions emerging from the surface of the virus particles (also called virions), which give them an appearance reminiscent of a crown. CoVs belong to the family of Coronaviridae, which is divided into two subfamilies. The main subfamily—Orthocoronavirinae—is subdivided into four genera, designated *Alphacoronavirus, Betacoronavirus, Gammacoronavirus* and *Deltacoronavirus*. CoVs generally have a limited host range and, with a few exceptions, cause either respiratory or enteric infections in mammals and birds. Until recently, six different human CoVs were distinguished, all of which primarily infect the respiratory tract (Tab. 1). Of these viruses, HCoV-229E, HCoV-HKU1, HCoV-OC43 and HCoV-NL63 are responsible for some 10–15% of the annual common colds [3]. Whereas infections with these viruses usually take a mild course this is not the case for infections with severe acute respiratory syndrome (SARS)-CoV and Middle East respiratory syndrome (MERS)-CoV, for which case-fatality rates of ~10 and ~35%, respectively, have been reported [4].

**Table 1:** Overview of currently known human coronaviruses

Coronavirus	First PubMed ID(s)—year / Country of origin	Genome size / GenBank Acc. No	Receptor	Disease
Alphacoronavirus HCoV-229E	4285768—1966 United States of America	27,317 nts AF304460	ANPEP	Endemic common colds
Betacoronavirus HCoV-HKU1	15613317—2005 China	29,926 nts AY597011	9-*O*-acetylated sialic acid	Endemic common colds
Betacoronavirus HCoV-OC43	5231356, 4298953—1967 United States of America	30,741 nts AY585228	9-*O*-acetylated sialic acid	Endemic common colds
Alphacoronavirus HCoV-NL63	15034574—2004 The Netherlands	27,553 nts AY567487	ACE2	Endemic common colds
Betacoronavirus SARS-CoV	12690092, 12690091, 12711465—2003 China	29,751 nts AY274119	ACE2	SARS epidemic 2002–2004
Betacoronavirus MERS-CoV	23075143, 15073334—2012 Saudi Arabia	30,119 nts JX869059	DPP4	MERS epidemic 2012-now
Betacoronavirus SARS-CoV‑2	31986261, 31978945, 32015508, 332015507—2020 China	29,903 nts NC_045512	ACE2	COVID-19 pandemic 2020–now

*nts* nucleotides, *ANPEP* alanyl aminopeptidase, *ACE2* angiotensin I converting enzyme 2, *DPP4* dipeptidyl peptidase 4

Recently, a new human CoV has emerged in Wuhan, People’s Republic of China, which appears genetically very closely related to several bat CoV isolates and somewhat more distantly to SARS-CoV [5, 6]. Based on phylogenetic considerations, the new virus was named SARS-CoV‑2, which stands for severe acute respiratory syndrome coronavirus 2 [7], and the accompanying disease was designated coronavirus disease 2019 (COVID-19). Like SARS-CoV and MERS-CoV, SARS-CoV‑2 is assumed to be a zoonotic virus with bats serving as reservoir hosts. Transmission of SARS-CoV and MERS-CoV from bats to humans likely occurred via intermediate hosts (presumably civet cats in the case of SARS-CoV and dromedary camels for MERS-CoV) to allow these viruses to adapt in a stepwise fashion to their new host species. Spillover of SARS-CoV‑2 from bats to humans probably also involved an intermediate host. Whether pangolins have acted as such, as initially proposed, is, however, doubtful [8]. The extraordinary ability of RNA viruses to adapt to changing selective pressures and to invade new hosts stems from the low fidelity with which they replicate their genome (i.e. genetic material) in combination with their high replication rate (i.e. short generation time). As a consequence, the genomes of RNA viruses are highly heterogeneous, consisting of dynamic populations of genetically related variants, also known as mutant swarms or quasispecies [9]. This not only allows RNA viruses to infect new host species but also to escape immune surveillance and to become resistant to antiviral drugs.

## Coronavirus life cycle

**Fig. 1:**
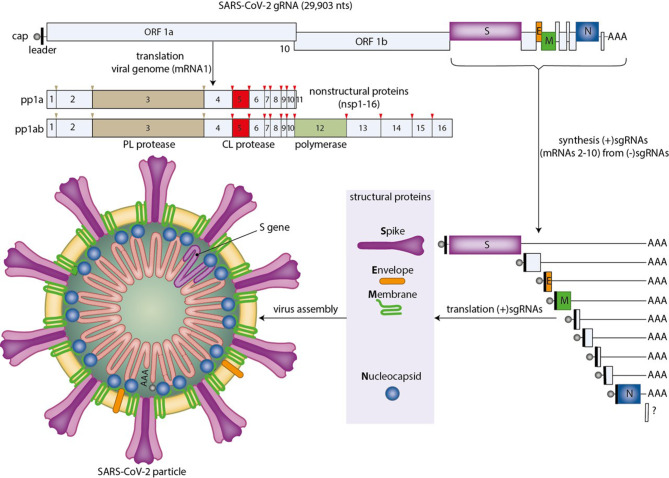
Schematic representations of SARS-CoV‑2 genome organisation, gene expression strategy and virion structure. See running text for explanation

**Fig. 2:**
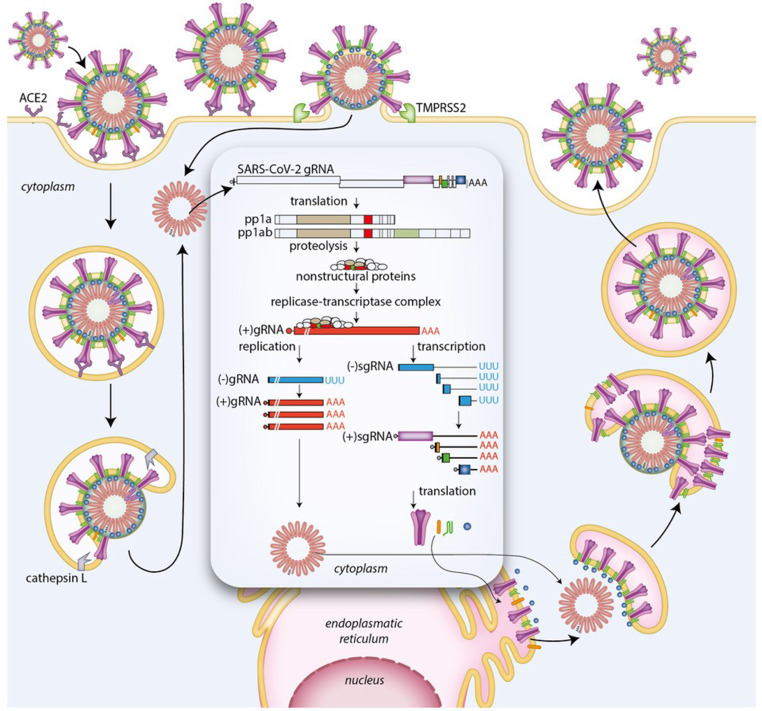
Schematic drawing of SARS-CoV‑2 life/infectious cycle. See running text for explanation. *ACE2* angiotensin I converting enzyme 2, *TMPRSS2* transmembrane serine protease 2

CoV particles are roughly spherical, have a diameter of 120–160 nm and consist of a core (also called nucleocapsid) surrounded by a protective coat or envelope (Fig. 1; [10]). The core contains the viral genome complexed with the nucleocapsid (N) protein. CoV genomes consist of a single RNA molecule of positive polarity with a length of ~26.4 to ~31.7 kilobases (kb). The envelope is composed of a lipid bilayer (like the one found at the surface of cells) in which several transmembrane proteins are inserted. For SARS-CoV‑2 the major envelope proteins are the spike (S) protein, the membrane (M) protein and the envelope (E) protein. The S protein is involved in the binding of the virus to target cells and has a crucial role in the penetration of these cells by mediating membrane fusion. After binding of SARS-CoV‑2 particles to its cellular receptor angiotensin I converting enzyme 2 (ACE2) and cleavage of the S protein by transmembrane serine protease 2 (TMPRSS2), the viral envelope fuses with the plasma membrane of the target cell, resulting in the delivery of the viral genome inside the cell (Fig. 2; [11, 12]). Alternatively, upon binding of their S protein to ACE2, SARS-CoV‑2 particles are taken up by the target cell in small vesicles called endosomes. Next, the S protein is cleaved by the endosomal protease cathepsin L, which initiates the fusion of the viral envelope with the lipid bilayer of the endosome, causing the release of the viral genome into the cytoplasm of the cell. A recent paper suggested that SARS may utilise CD147 (also known as basigin or EMMPRIN) as an alternative attachment receptor to enter target cells [13]. After the CoV genome, which is actually a very long mRNA [(+)gRNA], is set free inside the cell, it is immediately translated to produce two large polyproteins designated pp1a and pp1ab (Fig. 1). Synthesis of pp1ab requires the translation machinery to switch reading frame before encountering the stop codon of open reading frame (ORF) 1a by a process called ribosomal frameshifting. Autoproteolytic cleavage of pp1a and pp1ab produces 16 different nonstructural proteins (nsps), which together form the viral replicase complex [14]. This complex makes a complementary copy of the viral RNA genome called the antigenome [(−)gRNA], which serves as a template for the synthesis of new CoV genomes (replication) (Fig. 2). These (+)gRNA molecules, in turn, form the templates for the generation of a nested set of so-called subgenomic (sg) RNAs of negative polarity [(−)sgRNAs] with a common leader sequence complementary to the 5’ (i.e. left) end of the viral genome. These negative-strand sgRNAs are subsequently transcribed to produce a nested set of positive-strand sgRNAs (Figs. 1 and 2). Translation of these (+)sgRNAs yields the major structural proteins (i.e. the S, M, E and N protein in the case of SARS-CoV-2) and a number of accessory proteins [15, 16]. The accessory proteins of CoVs are involved in the modulation of different cellular processes for the benefit of the virus. Most accessory proteins perform multiple functions and several of them are incorporated into virus particles as minor structural proteins [17]. The N protein binds the newly synthesised positive-strand RNA genomes. The resulting ribonucleoprotein complexes (i.e. nucleocapsids) associate with membranes of the endoplasmic reticulum (ER)-Golgi intermediate compartment (ERGIC) in which the S, M and E proteins are inserted. Subsequently, virus particles are formed by budding of the capsids through the ERGIC membranes (Fig. 2). These virus particles are then transported in vesicles (i.e. exosomes) to the surface of the cell, where they fuse with the plasma membrane, leading to the release of the newly assembled virus particles in the extracellular space (Fig. 2). From there they can spread to new target cells to initiate additional rounds of virus production.

## COVID-19 pandemic

Since the first report on SARS-CoV‑2 on 31 December 2019 by the Wuhan Municipal Health Commission, the virus has spread rapidly across the globe, creating a pandemic with a colossal socioeconomic impact affecting all continents except Antarctica. As of 7 July 2020, John Hopkins University had registered 11,626,759 confirmed cases of COVID-19 and 538,190 COVID-19-related deaths, which would correspond to a global case-fatality rate of ~6%. Due to the limited testing capacity in many countries, especially at the beginning of the pandemic, and the existence of many asymptomatic and paucisymptomatic COVID-19 patients, the true incidence of SARS-CoV‑2 is presumably much higher. As a consequence, the true fatality rate will be lower too. Yet, excess mortality data indicate that the number of COVID-19-related deaths is also considerably higher than the reported death count (https://voxeu.org/article/excess-mortality-england-european-outlier-covid-19-pandemic), which may be caused by misdiagnosis and/or underreporting. For the well-characterised COVID-19 outbreak in February 2020 on the cruise ship Diamond Princess, case-fatality and infection-fatality rates for SARS-CoV‑2 of 2.6% and 1.3%, respectively, were calculated [18]. However, these figures may also not be fully representative of the global situation because of the relatively old age of the passengers, on the one hand, and the relatively high-quality care provided to the patients, on the other. Indeed, demographic differences as well as differences in health status, health care, COVID-19 treatment and cause of death assessment (i.e. did a person die with or die from COVID-19?), may explain the differences in reported case-fatality rates between different countries. By comparison, the global case-fatality rate associated with seasonal influenza epidemics is ~0.1%. The risk of COVID-19 hospital death is positively correlated with age, body-mass index and socioeconomic deprivation, e.g. in people ≥80 years of age an adjusted hazard ratio (HR) of 12.64 has been reported [19]. Males are ~2-fold more likely to die from COVID-19 than females and mortality in Caucasians is lower than in the other races [19, 20]. Recently, blood group has also been identified as a risk factor for acquiring COVID-19 with respiratory failure, i.e. blood group O and A are associated with, respectively, a lower and higher risk of acquiring severe COVID-19 than the other blood groups [21]. Most comorbidities are associated with a higher risk of COVID-19 hospital death, including cardiovascular disease, diabetes, (haematological) cancer, hypertension and respiratory disease [19, 20] although after adjustment for multiple variables the association with high blood pressure was lost and with chronic heart disease was rather weak (HR 1.27) [19]. This illustrates the fact that care should be taken in interpreting the results of univariate analyses.

## SARS-CoV-2 transmission

In the absence of a SARS-CoV‑2 vaccine and protective immunity resulting from infections with endemic human CoVs, current efforts to bring the pandemic to a halt are focusing on the reduction of the basic reproduction number (R_0_), which is defined as the expected number of secondary cases produced by a typical infected individual during the entire infectious period in a completely susceptible (i.e. non-immune) population and without any deliberate actions to reduce disease transmission. Based on early transmission dynamics in Wuhan, Li et al. estimated the R_0_ for SARS-CoV‑2 to be 2.2 (95% confidence interval (CI) 1.4–3.9) [22].

Since COVID-19 is primarily a respiratory infection, respiratory droplets produced (in decreasing numbers) by sneezing, coughing, singing, talking and breathing are the main sources of virus transmission.

A treacherous aspect of SARS-CoV‑2 infections is the presymptomatic transmission of the virus, which may have played an important role in the (rapid) spreading of the virus around the globe. By studying 77 transmission pairs and based on a mean incubation time of 5.2 (95% CI 4.1–7.0) days, He et al. inferred that infectiousness started from 2.3 (95% CI 0.8–3.0) days before symptom onset and peaked at 0.7 (95% CI −0.2–2.0) days before onset of illness to gradually decline afterwards [23]. This resulted in an estimated presymptomatic transmission rate of 44%, implying that containment measures based on isolation of virus shedders will only be effective if contact tracing includes the 2–3 days before symptom onset in the index case.

In several studies, SARS-CoV‑2 RNA has been demonstrated by reverse transcription-quantitative polymerase chain reaction (RT-qPCR) analysis (see below) in stool and sewage, and occasionally infectious virus has been recovered from faecal samples [24, 25]. Moreover, SARS-CoV‑2 has been shown to replicate in enterocytes of human small intestinal organoids [26], and the viral N has been detected by immunofluorescence microscopy in the cytoplasm of gastric, duodenal and rectal epithelial cells but not in oesophageal epithelial cells of endoscopic biopsies [27]. This raises the possibility of faecal transmission of SARS-CoV‑2, although formal evidence for this has not yet been obtained.

Recently, different species of companion and farm animals including cats, dogs, ferrets, hamsters and minks have been shown to be permissive to SARS-CoV‑2 infection (see, for example, [28]) and evidence has been obtained suggesting transmission of the virus from humans to these domesticated animals and vice versa (https://www.rivm.nl/en/novel-coronavirus-covid-19/pets).

For additional information on SARS-CoV‑2 transmission, see Appendix 1 of the Electronic Supplementary Material.

## Clinical manifestations

The outcome of SARS-CoV‑2 infections is highly variable, which may relate to several factors including (1) infectious dose, (2) general health of the infected individual and (3) appropriateness of his/her immune response. A large percentage (up to 81%) of people are unaware of being infected with SARS-CoV‑2 [29]. Analysis of 44,415 confirmed COVID-19 cases from China as of 11 February 2020 indicated that 81% had mild disease, 14% were severely ill and 5% developed critical illness, resulting in a case-fatality rate of 2.3% [30]. In mildly affected individuals the infection remains largely restricted to the upper respiratory tract, while in seriously ill patients it subsequently spreads to the lower respiratory tract and, from there, possibly to other organs. The most common (flu-like) symptoms of COVID-19 are chills/pyrexia, dry cough, dyspnoea and fatigue or myalgia. Other, less common, symptoms include expectoration, nasal congestion/rhinorrhoea, sore throat, headache, conjunctivitis, abdominal pain, diarrhoea, nausea or vomiting, ageusia, anosmia, skin rash and discoloration of fingers or toes. These symptoms are usually mild and develop gradually (reviewed in [4, 31, 32]). More seriously affected individuals suffer from pneumonia, accompanied by progressive dyspnoea, chest pain, haemoptysis, crackles and/or respiratory insufficiency. In the most severe cases of COVID-19, the pneumonia develops into acute respiratory distress syndrome (ARDS), which may lead directly to respiratory failure and is a major cause of COVID-19-related death. The development of ARDS is thought to largely result from the hypercytokinaemia (cytokine storm) induced by the virus and, together with secondary microbial infections (e.g. due to loss of intestinal epithelial barrier integrity), can culminate in sepsis and end-organ dysfunction [33, 34]. Interestingly, manifestations of COVID-19 in children are generally less severe than in adults. The precise reason(s) for this is/are unclear. Due to the higher incidence of respiratory infections, the innate immune system of children may be ‘trained’ and therefore better capable of mounting an efficient first line of defence against the virus, preventing the induction of a cytokine storm due to vigorous replication of SARS-CoV‑2 in the lower airways. Alternatively, in children replication of SARS-CoV‑2 could be suppressed by the presence of other respiratory viruses. Also, airway epithelial cells of children may be less permissive to SARS-CoV‑2, e.g. because of lower ACE2 expression levels [35].

The average incubation period of SARS-CoV‑2 is ~5 days with a 95% CI ranging from 2 to 14 days [22, 36]. In a study of 41 hospitalised COVID-19 patients, 22 developed dyspnoea with a median time from illness onset to dyspnoea of 8.0 days (interquartile range (IQR) 5.0–13.0 days). In the same study, medium times from onset of symptoms to hospital admission, ARDS, mechanical ventilation and admission to the intensive care unit (ICU) were 7.0 (IQR 4.0–8.0), 9.0 (IQR 8.0–14.0), 10.5 (IQR 7.0–14.0) and 10.5 (IQR 8.0-17.0) days, respectively [37]. In another study of 191 inpatients with laboratory-confirmed COVID-19, median times from illness onset to dyspnoea, ARDS, ICU admission and death or discharge were 7.0 (IQR 4.0–7.0), 12.0 (IQR 8.0–15.0), 12.0 (IQR 8.0–15.0) and 21.0 (IQR 17.0–25.0) days, respectively [38]. The median duration of virus shedding after onset of symptoms was 20.0 (IQR 16.0–23.0) days in this study.

Chest radiography of 64 Chinese COVID-19 patients with RT-qPCR confirmation, showed abnormal X‑ray images in 69% of the patients. In 6 of these patients, radiographic abnormalities were observed before testing positive for SARS-CoV‑2 RNA. Consolidation and ground-glass opacities were the dominant findings and mostly involved the periphery of the lower halves of both lungs [39]. Computed tomography (CT) scans of 81 confirmed COVID-19 patients before and ≤1 week, >1–2 weeks and >2–3 weeks after symptom onset revealed abnormalities in 2.8, 11.1, 13.0 and 12.1 of the 20 lung segments, respectively. CT image abnormalities rapidly evolved from predominantly focal unilateral to diffuse bilateral ground-glass opacities that progressed to or co-existed with consolidations within 1–3 weeks [40]. For more information on this topic, see [41].

While the respiratory tract is the primary target of SARS-CoV‑2, the clinical symptoms mentioned above show that other organs/organ systems, including the cardiovascular system, the digestive tract, the liver, the kidney, the brain and the eyes, can also be affected by COVID-19 [42–45]. Although SARS-CoV‑2 particles/components have been detected in, for example, endothelial cells, the digestive tract and the liver, not all extrarespiratory manifestations of COVID-19 are necessarily caused by direct viral injury but may also be the consequence of the hypoxaemia, (hyper)inflammatory response, neuroendocrine imbalance and other pathophysiological changes induced by the airway infection [43]. For information on the clinical management of (severe) COVID-19 see, for example, Nicola et al. [46] and Xie et al. [47]. Primarily intended for low- and middle-income countries, the Clinical Care for Severe Acute Respiratory Infection Toolkit of the World Health Organization is another excellent resource for managing adult and paediatric patients with severe forms of COVID-19 (https://www.who.int/publications/i/item/clinical-care-of-severe-acute-respiratory-infections-tool-kit).

## Immune response

A brief introduction to antiviral immune responses in general is provided in Appendix 1 of the Electronic Supplementary Material.

In the large majority of persons, infection with SARS-CoV‑2 is rapidly and efficiently controlled by the immune system and remains (largely) confined to the upper respiratory tract. As a consequence, these people develop no or only mild disease. However, in a small percentage of infected individuals the lower respiratory tract also becomes infected, accompanied by hyperinflammation and overactivation of the immune system, leading to excessive production of cytokines and accumulation of immune cells in the lungs. Due to the severe injury caused by the virus and by the immune system to the airway epithelial cells and the underlying endothelial cells, the alveolar-capillary barrier is broken, resulting in vascular leakage, alveolar oedema/collapse and ARDS. This may be followed by further clinical deterioration and ultimately cause death as a result of multi-organ damage/failure due to secondary SARS-CoV‑2 and bacterial infections and immune-mediated mechanisms.

Although detailed knowledge about the interactions between the innate immune system and SARS-CoV‑2 is still scarce, innate immune responses are thought to play an important role in limiting the viral infection (to the upper respiratory tract). Based on previous research on SARS-CoV and MERS-CoV and various animal models of virus-induced acute lung injury as well as haematological and biochemical laboratory findings in COVID-19 patients, the following scenarios can be envisioned [33, 34, 48, 49]. Infection of upper airway epithelial cells, recognition/uptake of SARS-CoV‑2 particles by dendritic cells and resident macrophages and activation of the complement system in the upper airways trigger the local production and release of pro-inflammatory cytokines and chemokines. These proteins induce chemotaxis of neutrophils, monocytes/macrophages, natural killer (NK) cells and lymphocytes to the site(s) of infection and participate in the activation of these immune cells. In the case of an adequate immune response, the virus infection is subsequently cleared by the concerted action of innate immune cells, the complement system, T and B lymphocytes and SARS-CoV-2-specific antibodies. However, when the immune response overshoots, a viscous cycle is induced of further pulmonary accumulation of immune cells, production of more pro-inflammatory factors and aggravated immune-mediated lung injury.

In patients with severe COVID-19, but less so in patients with mild disease, lymphopenia is commonly observed, with drastically reduced numbers of B cells, helper (i.e. CD4^+^) T cells (THs), cytotoxic (i.e. CD8^+^) T cells (CTLs) and NK cells. These patients also have an increased percentage of neutrophils and a decreased percentage of monocytes, eosinophils and basophils ([50] and references therein). In general, neutrophil count and neutrophil-to-lymphocyte ratio positively correlate with disease severity and a worse clinical outcome [51]. In addition, NK cells and CD8^+^ T cells show signs of functional exhaustion, the extent of which is directly proportional to disease severity. During recovery from COVID-19, the numbers of B cells, THs, CTLs and NK cells as well as exhaustion marker expression in cytotoxic lymphocytes normalise ([50] and references therein).

The specific B‑cell responses (i.e. antibody production) to SARS-CoV‑2 have been studied in detail by Long et al. [52] in 285 patients. At 2–4 days after onset of symptoms, SARS-CoV-2-specific immunoglobulin (Ig) G and/or IgM antibodies were detected in 6% of the serum samples. After 17–19 days all patients had SARS-CoV-2-specific IgGs, while virus-specific IgMs reached a peak of 94.1% at 20–22 days after symptom onset. During the first 3 weeks after onset of illness, SARS-CoV-2-specific IgG and IgM levels gradually increased. Severely diseased patients had significantly higher virus-specific IgG titres than mildly affected individuals at 2 weeks after symptom onset. The median day of seroconversion for both IgG and IgM was 13 days after onset of illness. Padoan and colleagues studied the kinetics of SARS-CoV-2-specific antibodies for 6 weeks after the onset of fever in 19 adult patients with RT-qPCR-confirmed COVID-19 [53]. Average SARS-CoV-2-specific IgA (i.e. mucosal antibody) and IgM levels above background started to be detected at 6 and 8 days after the onset of COVID-19, respectively. The average level of SARS-CoV-2-specific antibodies was higher for IgA than for IgM for the whole observation period. The average IgG titre peaked at day 20–22 and remained fairly constant for 1 month, while the average IgM level peaked at 10–12 days and gradually dropped thereafter.

Relatively little is known about the cellular immune response to SARS-CoV‑2. Using a large pool of peptides representing predicted T‑cell epitopes, SARS-CoV-2-specific CD8^+^ and CD4^+^ T lymphocytes were identified in ~70 and 100% of COVID-19 convalescent patients, respectively [54]. The majority of the THs and CTLs were directed against the highly expressed S, M and N proteins of SARS-CoV‑2. THs to the S protein were robust and showed a positive correlation with SARS-CoV‑2 IgG and IgA titres. Interestingly, SARS-CoV-2-reactive CD4^+^ T lymphocytes were detected in ~50% of unexposed individuals, indicative of possible cross-reactive T‑cell recognition between endemic CoVs and SARS-CoV‑2. In another study of 14 COVID-19 convalescent patients, variable numbers of nsp5-, S‑ and N‑specific T cells were detected and a direct correlation between the neutralising antibody titre and the strength of the N‑specific T‑cell response was found [55].

## Diagnosis

Diagnostic assays for COVID-19 fall into two categories: (1) tests to detect viral components, i.e. SARS-CoV‑2 RNA or protein; (2) assays to measure adaptive immunity (i.e. antibodies and T lymphocytes) against SARS-CoV‑2 infection [56, 57]. Although not specific, radiography to visualise lung disease, biomarker analysis to assess tissue/organ damage and inflammation, electron microscopy to visualise virus particles and virus infectivity assays to quantify functional virus particles can also aid in the diagnosis of COVID-19.

### Virological tests

RT-qPCR-based laboratory assays on upper or lower respiratory tract samples are presently the standard tests to detect ongoing SARS-CoV‑2 infections. RNA-virus detection by RT-qPCR is a multistep procedure involving the extraction of RNA from the clinical specimen, the preparation of DNA copies of the extracted RNA by an enzyme called reverse transcriptase, the logarithmic amplification of viral sequences in a so-called thermocycler using a thermostable DNA polymerase and virus-specific DNA primers and the real-time detection of the amplification products using fluorescence-based methods (see, for example, https://www.youtube.com/watch?v=ThG_02miq-4 for an illustrative movie).

The preferred specimens for the detection of SARS-CoV‑2 RNA by RT-qPCR are nasopharyngeal samples collected with a flocked swap and preserved in an appropriate transport medium. Besides in airway samples, SARS-CoV‑2 RNA is also frequently detected in faeces (reflecting infection of the gastrointestinal tract), occasionally in blood samples (indicative of viraemia) and rarely in urine.

**Fig. 3:**
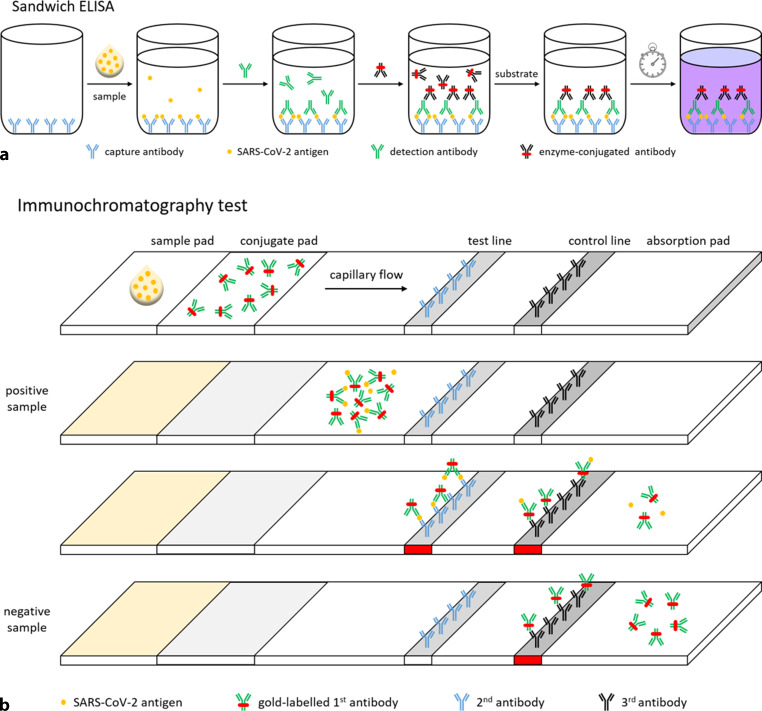
**a**, **b** Antigen detection methods. **a** Sandwich ELISA. **b** Immunochromatography test

An alternative method to confirm active infections is by a sandwich enzyme-linked immunosorbent assay (ELISA; Fig. 3a). This assay detects viral protein (i.e. antigen) in clinical specimens after lysis of the cells and virus particles in the sample. The resulting solution is applied to a well coated with a so-called capture antibody specific for one of the virion proteins. Next, an antibody recognising another epitope of the same virion protein (the so-called detection antibody) is applied to the well, followed by an enzyme-conjugated third antibody directed against the detection antibody. A colourless substrate is then added to the well, which can be converted by the enzyme into a coloured product that is detected with a colorimeter. The amount of coloured product that is generated correlates with the amount of virus present in the clinical specimen. Since a sandwich ELISA is a laborious procedure requiring multiple washing steps after each addition, which are usually performed automatically, this method is unsuitable for point-of-care testing. This has led to the development of immunochromatography strips for fast instrument-free testing employing capillary movement (Fig. 3b). These strips contain a sample pad for specimen application followed by a conjugate pad containing an antigen-specific antibody linked to, for example, colloidal gold or latex particles (first antibody). If the sample contains antigens, complexes are formed with the first antibody, which migrate to the so-called test line, where a fraction of these complexes are bound by another antigen-specific antibody covalently attached to the strip (second antibody), resulting in the formation of a coloured line. The remaining complexes and non-complexed first antibodies migrate further until they reach the so-called control line, which contains covalently attached antibodies recognising the first antibody (third antibody). This results in the formation of a second coloured line, irrespective of the presence of antigen in the clinical specimen.

In general, antigen detection assays are less sensitive than genome detection methods, resulting in a higher rate of false-negative results. Moreover, whereas RT-qPCR tests and sandwich ELISAs can provide a reasonable estimate of virus load, immunochromatography tests do not allow accurate quantitative analyses. In early infections, the amount of SARS-CoV‑2 in specimens from the upper part of the respiratory tract may be too low to be detected and repeated sampling may be necessary. Virological tests are not only instrumental in determining whether somebody is infected with SARS-CoV‑2 but also can be used for monitoring disease progression (by taking samples at different sites), to identify virus shedders and for taking de-isolation decisions.

### Serological tests

Different studies report somewhat different times after the onset of symptoms at which the first antibodies against SARS-CoV‑2 proteins are detected. Moreover, the extent and kinetics of the humoral immune response to SARS-CoV‑2 varies between individuals. This may relate to differences in infectious dose but also be the result of inherited and acquired differences in their immune system. In most studies, IgM, IgG and IgA antibodies directed against the SARS-CoV‑2 S and N proteins are first detected at roughly the same time, i.e. a few days after the onset of symptoms. IgM and IgG levels peak at 2–3 weeks after illness onset and drop faster afterwards for IgMs (see also above). Serological tests alone can thus not be used to establish with certainty that a person is infected with SARS-CoV‑2. The formats of antibody detection tests are similar to those of the antigen detection assays except that viral proteins (most S or N, but sometimes also whole virus lysates) serve as bait.

Serological tests are particularly useful in surveillance and containment programmes and for epidemic forecasting. They can also be used for assessment of immunity resulting from natural infection or active immunisation. However, it is not yet clear to what extent Ig levels correlate with protective immunity. In this respect, virus neutralisation assays (e.g. plaque reduction and tissue culture infectious dose assays) may be more informative. In these cell culture assays, virus is mixed with (different dilutions) of patient’s (or control) serum and added to susceptible cells to determine whether the serum contains antibodies that can inhibit virus infection.

A comprehensive overview of (1) diagnostic tests, (2) performance data of commercially available diagnostic assays, including information on their sensitivity and specificity, and (3) use case descriptions of diagnostic tests for COVID-19 can be found at https://www.finddx.org/covid-19/. See also Appendix 1 of the Electronic Supplementary Material.

## Treatment and prevention

In theory, SARS-CoV‑2 could be completely eradicated by strict isolation of all infectious sources similar to the extinction of a predator in the absence of prey. However, in practice such an approach seems unfeasible given (1) the extent of the virus’s spread and the huge socioeconomic impact of global containment measures and (2) the required intense and persevering surveillance using virological tests that can identify all virus shedders, including individuals with an asymptomatic SARS-CoV‑2 infection.

### Vaccines

**Fig. 4:**
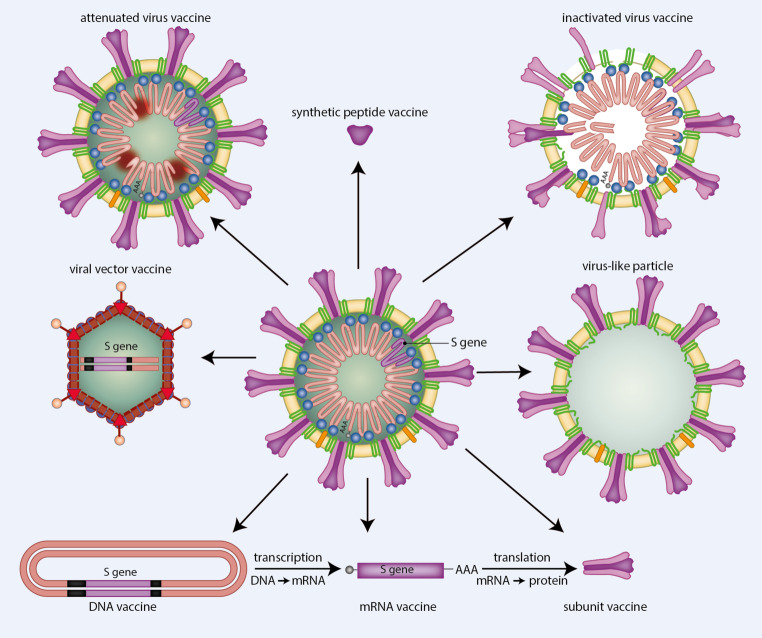
Overview of the different approaches to developing a vaccine for SARS-CoV‑2

This has created a huge incentive for the development of a safe and effective SARS-CoV‑2 vaccine (see https://docs.google.com/spreadsheets/d/16DbPhF9OD0MHHtCR12of6yUcfiRzP_-XGkynEbnipds/edit#gid=1804775590 for an overview) to block infection or, at least, mitigate the consequences of infection. Although there are currently no approved vaccines for human CoVs, the development of a vaccine for COVID-19 may benefit from (1) past and ongoing vaccine studies of SARS-CoV and MERS-CoV as well as (2) the successes and failures of animal CoV vaccines. This will, however, not obviate the need for extensive testing of candidate vaccines in appropriate animal models and clinical studies, especially since correlates of protection (e.g. neutralising antibody titres) may be different for different CoVs. The repertoire of SARS-CoV‑2 vaccines that is currently being developed comprises both replicating and non-replicating vaccines (Fig. 4; [58–60]). The non-replicating vaccines include (1) wild-type SARS-CoV‑2 particles inactivated by chemical treatment or high-energy radiation, (2) non-replicating viral vectors directing the expression of one or more SARS-CoV‑2 antigens, (3) virus-like particles (i.e. assemblies of one or more of the viral envelope proteins often with a lipid envelope), (4) mRNA or plasmid DNA encoding one or more viral proteins (in particular the S protein as the principal target of virus-neutralising antibodies), (5) subunit vaccines consisting of (parts of) the S protein in monomeric or trimeric form and (6) vaccines based on synthetic peptides representing viral antigenic determinants/epitopes. As these ‘dead’ vaccines are generally less immunogenic than ‘live’ vaccines, they are often administered together with an adjuvant to boost the immune response and to stimulate long-term immunological memory. The inclusion of an adjuvant may not be needed for the non-replicating viral vector- and nucleic-acid-based vaccines, as these vaccines will mediate de novo synthesis of SARS-CoV‑2 antigens inside target cells and therefore may induce a stronger immune response. One obvious advantage of especially inactivated virus- and nucleic-acid-based vaccines is that they can be produced in large amounts in a relatively short time. The replicating vaccines comprise (1) attenuated versions of SARS-CoV‑2 generated by serial passage in cultured cells or by genetic engineering and (2) replicating viral vectors encoding one or more SARS-CoV‑2 antigens. Although ‘live’ vaccines usually confer more durable and robust protection against disease than ‘dead vaccines’, their development time is generally much longer. Moreover, attenuated virus vaccines require extensive safety assessment to exclude the early appearance in vaccinees of variants/mutants that have regained their (full) disease-causing potential. More information about the pros and cons of the different types of vaccines and their effects in pre(clinical) studies can be found in various recent reviews [58–60].

A COVID-19 vaccine does not necessarily have to contain/express SARS-CoV‑2 proteins. Since innate immune responses represent the first line of defence against invading pathogens, boosting these responses by applying live vaccines directed against, for example, tuberculosis, poliovirus or measles virus may reduce the susceptibility to SARS-CoV‑2 infections and the severity of COVID-19 [48].

Despite the urgent need for a SARS-CoV‑2 vaccine, the safety aspects should not be neglected. One potential danger is that a SARS-CoV‑2 vaccine induces (poorly neutralising) antibodies, which facilitate subsequent virus uptake by myeloid cells via antibody or complement receptors present at their surface. This antibody-dependent enhancement of infection has previously been observed for feline infectious peritonitis virus (FIPV) in cats vaccinated with a recombinant poxvirus expressing the FIPV S protein [61–63]. Another risk is the induction, by vaccination, of a skewed type 2 TH (Th2) response, resulting in Th2-driven immunopathology accompanied by pulmonary eosinophilia, as observed with certain experimental SARS-CoV and MERS-CoV vaccines [62, 63]. Moreover, since studies on endemic human CoVs have suggested immunity against these viruses to be short-lived [64], it will be important to establish the duration of protection from disease following vaccination against COVID-19. Finally, one has to consider the possibility that a vaccine based on current SARS-CoV‑2 variants will provide no or only partial protection against future strains of the virus or other novel human CoVs. Nonetheless, active immunisation using an effective vaccine in combination with a rigorous surveillance and containment programme can lead to the complete eradication of a viral disease, as demonstrated for smallpox. Which type(s) of vaccine will eventually be used to combat COVID-19 will be determined by a combination of factors including safety, efficacy and time-to-market introduction.

### Antivirals

**Fig. 5:**
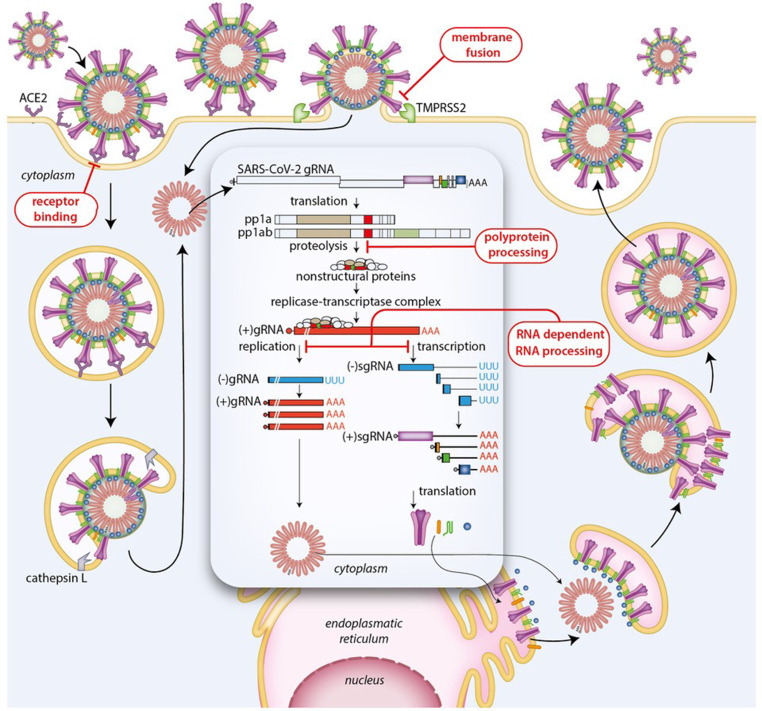
Overview of potential targets for the development of antiviral drugs for COVID-19. *ACE2* angiotensin I converting enzyme 2, *TMPRSS2* transmembrane serine protease 2

Rather than trying to prevent the development of SARS-CoV-2-related sickness by arming the immune system through vaccination, another strategy consists of thwarting the virus once an infection has revealed itself by the administration of antiviral drugs to the patient. Due to (1) the lack of approved drugs directed against human CoVs and (2) the long times involved in the development and evaluation of new antiviral agents, the main focus is currently on repositioning existing drugs designed for other (viral) diseases to treat COVID-19 patients [65]. In addition, unapproved drugs showing antiviral activity in animal models of SARS-CoV and MERS-CoV are being tested for the ability to inhibit SARS-CoV‑2 replication. Mechanistically, anti-SARS-CoV‑2 drugs exert their beneficial effect by inhibiting crucial steps in the viral life cycle like receptor binding, envelope fusion, polyprotein processing and RNA-dependent RNA synthesis (Fig. 5; [60, 63]).

Recombinant soluble human ACE2 (sACE2) is being evaluated for its ability to block the interaction between SARS-CoV‑2 and target cells. sACE2 could also compensate for the SARS-CoV-2-related reduction of surface ACE2 levels and consequential disturbance of angiotensin II/angiotensin-(1–7) balance [66] and thereby help to preserve pulmonary vascular integrity. S‑protein-specific neutralising monoclonal antibodies can either interfere with the binding of the virus to target cells or prevent fusion of the viral envelope with the plasma membrane or the membrane of endosomes. Merging of viral and cellular membranes and subsequent release of the viral genome into the host cell cytoplasm may also be inhibited using peptidomimetic fusion inhibitors or the influenza virus entry inhibitor arbidol (umifenovir). Several studies have also suggested a possible beneficial effect of the antimalarial drugs chloroquine and hydroxychloroquine on COVID-19 progression, which may at least in part be related to their ability to inhibit endosomal acidification and thereby membrane fusion of internalised virus particles [67, 68]. However, (hydroxy)chloroquine is known to cause QTc prolongation, which may result in a torsades de pointes type of ventricular tachycardia. Apart from using monoclonal antibodies for passive immunisation purposes (blood-group-matched) convalescent plasma of recovered COVID-19 patients could also be employed to protect newly infected individuals from developing severe disease [69].

SARS-CoV‑2 replication can also be inhibited by treatment with nucleoside analogues (usually administered as prodrug) that interfere with the RNA-dependent RNA synthesis needed to amplify the viral genome and to produce the subgenomic mRNAs. Nucleoside analogues under current investigation for the treatment of COVID-19 include ribavirin, favipiravir (T-705) and remdesivir (GS-5734).

Other pharmacological targets could be nsp3 and nsp5, the papain- and chymotrypsin-like proteases encoded by ORF 1a and involved in the processing of the SARS-CoV‑2 polyproteins that produce the viral replication and transcription complexes (Figs. 1 and 2). Accordingly, clinical trials have been initiated with the human immunodeficiency (HIV) protease inhibitor combination lopinavir/ritonavir. The in vivo effectiveness of these drugs against SARS-CoV‑2 remains to be seen as the HIV protease belongs to the family of aspartyl proteases and nsp3 and nsp5 are cysteine proteases.

A highly specific way to inhibit SARS-CoV‑2 would be by RNA interference. This method is based on the delivery to virus-infected/target cells of small (modified) RNA molecules (so-called small interfering RNAs or siRNAs) that are binding/complementary to the viral genome and promote its degradation. By simultaneous treatment of COVID-19 with multiple siRNAs targeting different parts of the SARS-CoV‑2 genome, it will be very hard for the virus to escape from this antiviral mechanism by changing its genetic code through mutation.

An alternative to the inhibition of viral proteins/processes is the suppression of host cell factors/processes required by the virus. Obvious targets are the SARS-CoV‑2 attachment receptor ACE2 and the host proteases involved in the processing of the S protein to render it fusion competent. However, care should be taken that antagonists of these host proteins do not interfere with vital physiological processes. Early activation of cellular defence systems could also be an effective means to suppress SARS-CoV‑2 replication. One possibility would be to treat patients with interferons, as these cytokines play an important role in the (1) development of early innate immune responses to viral infections, (2) activation of subsequent adaptive immune responses and (3) dampening of immunopathogenic mechanisms [70]. Support for this approach is provided by the finding that SARS-CoV and MERS-CoV counteract interferon signalling by multiple mechanisms and that replication of these viruses can be inhibited by interferons [15, 63, 71]. There may, however, also be a downside to treatment of COVID-19 with interferons given the recent finding that they can increase ACE2 expression in human airway epithelial cells [72].

In general, in patients at risk of developing severe disease following infection with SARS-CoV‑2, antiviral drug therapy should be started as soon as possible to minimise virus-induced damage of airway epithelium and to avoid the subsequent development of hypercytokinaemia. In addition, COVID-19 patients receiving antiviral drugs must be closely monitored for possible adverse effects and potential interactions with other drugs should be reckoned with. Similar to what has been found for HIV and hepatitis C virus, combining different antiviral drugs may have additive or synergistic effects. Antiviral drug cocktails will also lower the risk of the selection of SARS-CoV‑2 variants with mutations that resist antiviral drug therapy. Antiviral drugs may also be employed as prophylactics to prevent infection or to reduce virus shedding and thereby the risk of virus spreading.

### Immunomodulators

COVID-19 severity and COVID-19-associated mortality are positively correlated with the serum levels of (1) positive acute phase proteins (e.g. C‑reactive protein (CRP), ferritin and the fibrin degradation product D‑dimer), (2) inflammatory cytokines (e.g. interferon γ (INFγ), interleukin (IL) 1β, 6 and 7 and tumour necrosis factor α (TNFα)) and chemokines (e.g. C‑C motif chemokine 2 (CCL2), also known as monocyte chemoattractant protein 1 (MCP1); and C‑X‑C motif chemokine 10 (CXCL10), also known as interferon gamma-induced protein 10 (IP-10)) and (3) with the neutrophil-to-lymphocyte ratio [33, 37]. This lends support to the idea that SARS-CoV-2-induced hyperinflammation is a major contributor to COVID-19 pathogenesis. Accordingly, multiple clinical trials have been initiated to investigate the effect of immunomodulation on the course of COVID-19 [33, 60, 73]. In view of the essential role of both innate and adaptive immune responses in the clearance of (corona)viral infections, timing and dosing of the immunomodulatory therapy will have a large impact on the outcome of these studies. This is nicely illustrated by the fact that both inhibition and stimulation of granulocyte-macrophage colony-stimulating factor are considered as therapy for COVID-19 patients [33]. Other targets of immunomodulation include the IL1β, IL6, INFγ and TNFα signalling pathways. Inhibition of these signalling pathways is in most cases accomplished by therapeutic antibodies directed against the cytokine itself or its receptor. In addition, small molecule inhibitors of C‑C chemokine receptor type 2 and 5, the IL1 receptor and Janus kinase (JAK)-signal transducer and activator of transcription protein (STAT) signalling are being evaluated in clinical studies. Other immunomodulatory therapies that are currently being investigated include the administration of mesenchymal stem cells, NK cells, high doses of intravenous immunoglobulins (IVIG) or recombinant human surfactant protein D and the inhibition, by a small molecule drug and monoclonal antibody, of the processing of complement factors C3 and C5, respectively [74]. The use of glucocorticoids to curtail SARS-CoV-2-induced hyperinflammation is still highly controversial [75], while there are some indications for a suppressive effect of statins on the likelihood of developing COVID-19 symptoms in the elderly [76].

A comprehensive overview of drugs under development for COVID-19 can be found at https://docs.google.com/spreadsheets/d/16DbPhF9OD0MHHtCR12of6yUcfiRzP_-XGkynEbnipds/edit#gid=1206197573. When using antivirals or immunomodulators in the treatment of COVID-19, one should be vigilant for drug-induced organ toxicity, especially of the liver, brain, heart and kidney, and for (ventricular) arrhythmias.

### Anticoagulants

Multiple recent studies have identified venous and arterial thrombosis secondary to SARS-CoV‑2 infection as a major complication of COVID-19, which likely contributes to its relatively high mortality rate [77, 78]. The pathophysiological mechanisms involved in the COVID-19-associated coagulopathy are incompletely understood. Factors that may contribute to the thrombophilia observed in severely ill COVID-19 patients include the following: (1) a disturbed balance between pro- and anticoagulant activities due to excessive production of proinflammatory cytokines, activation of complement, formation of neutrophil extracellular traps and activation of platelets; (2) inflammation-related endothelial activation; (3) death of SARS-CoV-2-infected endothelial cells; (4) endothelial dysfunction caused by unbalanced angiotensin II-angiotensin II type‑1 receptor signalling; (5) formation of prothrombotic antiphospholipid antibodies; (6) immobility-associated reduction of blood flow; (7) hypoxia due to respiratory impairment resulting from SARS-CoV-2-induced lung injury [79–81]. Based on the high incidence of venous thromboembolism, myocardial infarction and stroke among COVID-19 patients and the mortality-reducing effect of empirical therapeutic anticoagulation with heparin in COVID-19 patients with a sepsis-induced coagulopathy score ≥4 [82], clinical trials have been initiated to investigate the impact of (different doses) of antithrombotics on disease outcome [79]. In addition, recommendations have been formulated for (tailored) prophylactic or therapeutic anticoagulation therapy in specific patient groups (see, for example, [80, 83]). In COVID-19 patients receiving antiviral therapy possible interactions with anticoagulants and antiplatelet drugs could occur, which preclude the use of certain drug combinations or require dose adjustments. If use of pharmacological antithrombotics is contraindicated, mechanical therapy could be considered. As heparin not only inhibits blood clotting but also possesses anti-inflammatory and anti-arrhythmic effects and may block SARS-CoV‑2 host-cell interactions, it could benefit COVID-19 patients in multiple ways [74].

## Cardiovascular manifestations

As already mentioned above, albeit patients with pre-existing cardiovascular disease (CVD) may not be more susceptible to contracting SARS-CoV‑2 than healthy individuals, they have a higher chance of developing severe COVID-19 and are more likely to die from SARS-CoV‑2 infection. The higher COVID-19-related mortality of CVD patients may be directly related to or coincident with their cardiovascular comorbidities. Although COVID-19 primarily disrupts the respiratory system, the disease frequently affects the cardiovascular system as well, especially in more severe cases. The currently available data suggest that some 20–30% of hospitalised COVID-19 patients display cardiac complications [84], and in a recent study of 671 severely ill COVID-19 patients acute myocardial injury and acute heart failure were identified as death-related complications in 30.6% and 19.4% of the 62 deceased patients, respectively [85]. Of these patients, 98.4% had ARDS and 90.3% suffered from acute respiratory failure. A small percentage of hospitalised COVID-19 patients develop cardiac disease in the absence of marked pulmonary illness [86].

The cardiovascular complications of COVID-19 include acute myocardial injury, heart failure with or without cardiogenic shock, pericardial effusion with or without tamponade, arrhythmias and sudden cardiac death and thrombosis of small and large blood vessels [31, 87].

The most common cardiac complication of COVID-19 is acute myocardial injury, as evinced by increased plasma levels of cardiac troponin and echo-/electrocardiographic abnormalities. In a retrospective study of 416 inpatients, 19.7% displayed cardiac injury defined as high-sensitivity cardiac troponin I (hs-cTnI) levels above the 99th percentile of a healthy reference population. The mortality rate among patients with elevated hs-cTnI levels was 51.2% versus 4.5% in patients without myocardial injury [88]. Moreover, disease severity and risk of death were positively correlated with hs-cTnI levels, even after controlling for other comorbidities. Interestingly, in another study, plasma troponin T levels demonstrated a high and significantly positive linear correlation with plasma high-sensitivity CRP levels and N‑terminal pro-brain natriuretic peptide levels [89], suggesting a link between cardiac injury, systemic inflammation and myocardial wall stress. Multiple mechanisms may be responsible for the myocardial injury observed in COVID-19 patients. It could be the result of atherosclerotic plaque erosion/rupture (i.e. obstructive coronary artery disease) induced by COVID-19-related hypoxaemia, systemic hyperinflammation or angiotensin-2/angiotensin-(1–7) misbalance [66] leading to type 1 myocardial infarction. The myocardial injury could also be caused by virus- or immune-mediated myocarditis, stress-induced cardiomyopathy or oxygen supply and demand mismatch resulting in type 2 myocardial infarction (i.e. myocardial infarction without acute coronary syndrome), possible linked to respiratory insufficiency and/or sepsis. Sepsis and the sepsis-related cytokine storm together with myocardial injury and ischaemia are likely involved in the occurrence of cardiac arrhythmias in COVID-19 patients. The cytokine storm is also thought to be largely responsible for the hypercoagulable state and consequential disseminated intravascular coagulation /thrombotic microangiopathy and venous thrombosis including pulmonary embolism often seen in (severely ill/deceased) COVID-19 patients. Other factors contributing to the coagulopathy could be endothelial damage due to (1) the hyperinflammatory response typical of severe COVID-19 cases, (2) disturbance of the angiotensin-2/angiotensin-(1–7) balance and (3) SARS-CoV‑2 infection of endothelial cells [81].

More extensive discussions about the cardiovascular aspects of COVID-19 are provided in some recent reviews [87, 90, 91]. For information on how to manage the various cardiovascular complications of the COVID-19 pandemic, see [92, 93] and the guidance provided by the European Society of Cardiology https://www.escardio.org/static_file/Escardio/Education-General/Topic%20pages/Covid-19/ESC%20Guidance%20Document/ESC-Guidance-COVID-19-Pandemic.pdf.

## Conclusion

After SARS-CoV and MERS-CoV, SARS-CoV‑2 is the third zootic betacoronavirus of the 21st century that imposes a serious threat to human health. The outcome of a SARS-CoV‑2 infection is highly variable and dependent on multiple factors, including SARS-CoV‑2 genotype, infectious dose, genetic background, health status, immunological readiness and age. While COVID-19 is primarily a respiratory disease and mostly has a mild course, in severe cases other organs are also affected, including the heart and vasculature. To what extent the cardiovascular manifestations of COVID-19 are due to direct viral injury of cardiovascular cells or are an indirect consequence of the extensive lung pathology and accompanying (hyper)inflammation remains to be determined. The COVID-19 pandemic has once again alerted us to the high adaptive potential of RNA viruses and the downsides of globalisation. At the same time, international collaboration between medical professionals, scientists and politicians will be instrumental in tackling the current and foreseeable future (corona)virus outbreaks.

## Caption Electronic Supplementary Material

The Electronic Supplementary Material consists of extended versions of the chapters SARS-CoV-2 transmission, Immune response and Diagnosis.
