# Supplementary material for: SARS-CoV-2/COVID-19: a primer for cardiologists
Source: Neth Heart J. 2020 Jul 15;28(7-8):366–83. doi: 10.1007/s12471-020-01475-1 (PMC7360901; doi:10.1007/s12471-020-01475-1)
Supplement: Supplementary file 1 — The Electronic Supplementary Material consists of extended versions of the chapters SARS-CoV-2 transmission, Immune response and Diagnosis. [file 12471_2020_1475_MOESM1_ESM.docx]

**Caption Electronic Supplementary Material**

**Appendix A:**

**SARS-CoV-2 transmission**

In the absence of a severe acute respiratory syndrome coronavirus 2 (SARS-CoV-2) vaccine and protective immunity resulting from infections with endemic human coronaviruses (CoVs), current efforts to bring the pandemic to a halt are focusing on the reduction of the basic reproduction number (R_0_), which is defined as the expected number of secondary cases produced by a typical infected individual during the entire infectious period in a completely susceptible (*i.e.* non-immune) population and without any deliberate actions to reduce disease transmission. Based on early transmission dynamics in Wuhan, Li et al. estimated the R_0_ for SARS-CoV-2 to be 2.2 [95% confidence interval (CI) 1.4-3.9] [1]. For comparison, the R_0_ for measles virus, poliovirus and seasonal influenzavirus range from 12-18, 5-7 and 0.9-2.1, respectively. Moreover, there seems to be a high degree of interindividual variation in the ability to spread SARS-CoV-2 with a small fraction of infected persons, the so-called superspreaders, being responsible for a large percentage of secondary transmissions [2]. To limit the spread of SARS-CoV-2, hand hygiene and cough and sneeze etiquette, the use of personal protective equipment, social distancing measures, travel restrictions and (self-)isolation of possible/confirmed SARS-CoV-2-infected individuals have been implemented in most of the affected countries.

Since coronavirus disease 2019 (COVID-19) is primarily a respiratory infection, respiratory droplets produced (in decreasing numbers) by sneezing, coughing, singing, talking and breathing are the main sources of virus transmission. As large droplets remain in the air for only a short time and travel only short distances, they mainly cause infection by touching a contaminated surface or by entering the spray zone of a virus shedder (contact transmission). Small droplets (*i.e.* aerosols) stay in the air for much longer periods and can travel long distances and therefore may cause airborne transmission of SARS-CoV-2, albeit this is still a heavily debated issue [3].

A treacherous aspect of SARS-CoV-2 infections is the presymptomatic transmission of the virus, which may have played an important role in the (rapid) spreading of the virus around the globe. By studying 77 transmission pairs and based on a mean incubation time of 5.2 (95% CI 4.1-7.0) days, He et al. inferred that infectiousness started from 2.3 (95% CI 0.8-3.0) days before symptoms onset and peaked at 0.7 (95% CI -0.2-2.0) days before onset of illness to gradually decline afterwards [4]. This resulted in an estimated presymptomatic transmission rate of 44%, implying that containment measures based on isolation of virus shedders will only be effective if contact tracing includes the 2-3 days before symptom onset in the index case.

While He et al. did not find a correlation between virus load (as determined by reverse transcription-quantitative polymerase chain reaction (RT-qPCR) analysis of throat swabs) and disease severity, Liu et al. found SARS-CoV-2 RNA in nasopharyngeal swabs to be present in higher amounts and for longer times in patients with severe COVID-19 than in mildly affected individuals [5]. Also relevant for the risk of infection with SARS-CoV-2 is the finding that the virus “survives” for relative long periods on glass, stainless steel, banknotes, plastic and surgical masks [6].

In several studies, SARS-CoV-2 RNA has been demonstrated by RT-qPCR analysis (see below) in stool and sewage, and occasionally infectious virus has been recovered from faecal samples [7,8]. Moreover, SARS-CoV-2 has been shown to replicate in enterocytes of human small intestinal organoids [9], and the viral N has been detected by immunofluorescence microscopy in the cytoplasm of gastric, duodenal and rectal epithelial cells but not in oesophageal epithelial cells of endoscopic biopsies [10]. This raises the possibility of faecal transmission of SARS-CoV-2, although formal evidence for this has not yet been obtained.

Occasionally, newborns of mothers diagnosed with COVID-19 have tested positive for SARS-CoV-2 [11], which may be the result of vertical virus transmission. However, none of the studies performed thus far has provided virological evidence for the intrauterine transmission of SARS-CoV-2 from mothers to children. It thus seems likely that the SARS-CoV-2-positive neonates mentioned above got infected via perinatal or postnatal transmission.

Recently, different species of companion and farm animals including cats, dogs, ferrets, hamsters and minks have been shown to be permissive to SARS-CoV-2 infection (see, for example, [12]) and evidence has been obtained suggesting transmission of the virus from humans to these domesticated animals and *vice versa* (<https://www.rivm.nl/en/novel-coronavirus-covid-19/pets>).

**Immune response**

Similar to other viral pathogens, the immune response to SARS-CoV-infections consist of a fast innate immune response followed by a specific acquired/adaptive immune response. The innate immune response is induced by certain (1) (conserved) microbial signatures designated pathogen-associated molecular patterns (PAMPs) and (2) factors released by injured/dying cells called damage-associated molecular patterns (DAMPs). The innate immune systems’ main functions are to (1) induce an inflammatory response allowing blood cells and proteins to enter the site(s) of infection, (2) establish an antiviral state in non-infected cells, (3) phagocytose viral particles (macrophages and neutrophils), (4) kill virus-infected cells (natural killer cells and complement) and (5) stimulate adaptive immunity. These functions are tightly regulated by cytokines released by phagocytes, infected cells and some other cell types after activation of DAMP/PAMP receptors as well as by certain complement factors. The adaptive immune response is mediated by B lymphocytes and T lymphocytes. B lymphocytes are the major cell type involved in the so-called humoral immune response. After their stimulation, B lymphocytes proliferate and subsequently differentiate into plasma cells that produce large amount of virus-specific antibodies. These antibodies can neutralize SARS-CoV-2 by interfering with receptor binding or membrane fusion or opsonize virus particles to facilitate their clearance by phagocytes but they can also activate complement and natural killer cells. T lymphocytes are responsible for the so-called cellular immune response and can be divided in (CD4^+^) helper T cells (THs) and (CD8^+^) cytotoxic T cells (CTLs). THs are involved in the activation of macrophages, B lymphocytes and CTLs. The main function of CTLs is to kill virus-infected cells. Small numbers of B and T lymphocytes differentiate into memory cells, which allows the host to initiate a quick and strong adaptive immune response after reinfection.

In the large majority of persons, infection with SARS-CoV-2 is rapidly and efficiently controlled by the immune system and remains (largely) confined to the upper respiratory tract. As a consequence, these people develop no or only mild disease. However, in a small percentage of infected individuals the lower respiratory tract also becomes infected, accompanied by hyperinflammation and overactivation of the immune system, leading to excessive production of cytokines and accumulation of immune cells in the lungs. Due to the severe injury caused by the virus and by the immune system to the airway epithelial cells and the underlying endothelial cells, the alveolar-capillary barrier is broken, resulting in vascular leakage, alveolar oedema/collapse and acute respiratory distress syndrome (ARDS). This may be followed by further clinical deterioration and ultimately cause death as a result of multi-organ damage/failure due to secondary SAR-CoV-2 and bacterial infections and immune-mediated mechanisms.

Although detailed knowledge about the interactions between the innate immune system and SARS-CoV-2 is still scarce, innate immune responses are thought to play an important role in limiting the viral infection (to the upper respiratory tract). Based on previous research on SARS-CoV and MERS-CoV and various animal models of virus-induced acute lung injury as well as haematological and biochemical laboratory findings in COVID-19 patients, the following scenarios can be envisioned [13,14,15,16]. Infection of upper airway epithelial cells, recognition/uptake of SAR-CoV-2 particles by dendritic cells and resident macrophages and activation of the complement system in the upper airways trigger the local production and release of pro-inflammatory cytokines and chemokines (via PAMP/DAMP signalling). These proteins induce chemotaxis of neutrophils, monocytes/macrophages, natural killer (NK) cells and lymphocytes to the site(s) of infection and participate in the activation of these immune cells. In the case of an adequate immune response, the virus infection is subsequently cleared by the concerted action of innate immune cells, the complement system, T and B lymphocytes and SARS-CoV-2-specific antibodies. However, when the immune response overshoots, a viscous cycle is induced of further pulmonary accumulation of immune cells, production of more pro-inflammatory factors and aggravated immune-mediated lung injury.

In patients with severe COVID-19, but less so in patients with mild disease, lymphopenia is commonly observed, with drastically reduced numbers of B cells, CD4^+^ T cells, CD8^+^ T cells and NK cells. These patients also have an increased percentage of neutrophils and a decreased percentage of monocytes, eosinophils and basophils ([17] and references therein). In general, neutrophil count and neutrophil-to-lymphocyte ratio positively correlate with disease severity and a worse clinical outcome. In addition, NK cells and CD8^+^ T cells show signs of functional exhaustion, the extent of which is directly proportional to disease severity. During recovery from COVID-19, numbers of B cells, THs, CTLs and NK cells and exhaustion marker expression in cytotoxic lymphocytes normalize ([17] and references therein).

The specific B-cell responses (*i.e.* antibody production) to SARS-CoV-2 have been studied in detail by Long et al. [18] in 285 patients. At 2-4 days after onset of symptoms, SARS-CoV-2-specific immunoglobulin (Ig) G and/or IgM antibodies were detected in 6% of the serum samples. After 17-19 days all patients had SARS-CoV-2-specific IgGs, while virus-specific IgMs reached a peak of 94.1% at 20-22 days after symptom onset. During the first 3 weeks after onset of illness, SARS-CoV-2-specific IgG and IgM levels gradually increased. Severely diseased patients had significantly higher virus-specific IgG titres than mildly affected individuals at 2 weeks after symptom onset. The median day of seroconversion for both IgG and IgM was 13 days after onset of illness. Padoan and colleagues studied the kinetics of SARS-CoV-2-specific antibodies for 6 weeks after the onset of fever in 19 adult patients with RT-qPCR-confirmed COVID-19 [19]. Average SARS-CoV-2-specific IgA (*i.e.* mucosal antibody) and IgM levels above background started to be detected at 6 and 8 days after the onset of COVID-19, respectively. The average level of SARS-CoV-2-specific antibodies was higher for IgA than for IgM for the whole observation period. The average IgG titre peaked at day 20-22 and remained fairly constant for 1 month while the average IgM level peaked at 10–12 days and gradually dropped thereafter.

Relatively little is known about the cellular immune response to SARS-CoV-2. Using a large pool of peptides representing predicted T-cell epitopes, SARS-CoV-2-specific CD8^+^ and CD4^+^ T lymphocytes were identified in ~70 and 100% of COVID-19 convalescent patients, respectively [20]. The majority of the THs and CTLs were directed against the highly expressed spike (S), membrane (M) and nucleocapsid (N) proteins of SARS-CoV-2. THs to the S protein were robust and showed a positive correlation with SARS-CoV-2 IgG and IgA titres. Interestingly, SARS-CoV-2-reactive CD4^+^ T lymphocytes were detected in ~50% of unexposed individuals indicative of possible cross-reactive T cell recognition between endemic CoVs and SARS-CoV-2. In another study of 14 COVID-19 convalescent patients, variable number of nsp5-, S- and N-specific T cells were detected and a direct correlation between the neutralizing antibody titre and the strength of the N-specific T cell response was found [21].

**Diagnosis**

Diagnostic assays for COVID-19 fall in two categories: (1) tests to detect viral components, *i.e.* SARS-CoV-2 RNA or protein; (2) assays to measure adaptive immunity (*i.e.* antibodies and T lymphocytes) against SARS-CoV-2 infection [22,23]. Although not specific, radiography to visualize lung disease, biomarker analysis to assess tissue/organ damage and inflammation, electron microscopy to visualize virus particles and virus infectivity assays to quantify functional virus particles can also aid in the diagnosis of COVID-19.

*Virological tests*

RT-qPCR-based laboratory assays on upper or lower respiratory tract samples are presently the standard tests to detect ongoing SARS-CoV-2 infections. RNA-virus detection by RT-qPCR is a multistep procedure involving the extraction of RNA from the clinical specimen, the preparation of DNA copies of the extracted RNA by an enzyme called reverse transcriptase, the logarithmic amplification of viral sequences in a so-called thermocycler using a thermostable DNA polymerase and virus-specific DNA primers and the real-time detection of the amplification products using fluorescence-based methods (see, for example, <https://www.youtube.com/watch?v=ThG_02miq-4> for an illustrative movie). In the case of SARS-CoV-2, common practice is to use two primer pairs targeting different (conserved) parts of the viral genome. While most assays are highly specific for SARS-CoV-2, in some tests one of the primer pairs is designed to recognize genetic material of all known human CoVs.

The preferred specimens for the detection of SARS-CoV-2 RNA by RT-qPCR are nasopharyngeal samples collected with a flocked swap and preserved in an appropriate transport medium. Besides in airway samples, SARS-CoV-2 RNA is also frequently detected in faeces (reflecting infection of the gastrointestinal tract), occasionally in blood samples (indicative of viraemia) and rarely in urine.

Given the high transmissibility of the virus, processing of the samples should be done in a biosafety level 2 facility by personnel wearing appropriate personal protection. Traditional RT-qPCR is a cyclic process involving repeated changes of temperature to allow multiple rounds of (1) unwinding of the DNA template, (2) binding of the primers to the unwound DNA and (3) DNA synthesis through primer extension.

Recently, various isothermal nucleic acid amplification methods for the detection of SARS-CoV-2 copy DNA have been developed obviating the need for a thermocycler. Moreover, CRISPR/Cas technology has been incorporated in test designs (see <https://blog.addgene.org/sars-cov-2-covid-19-detection-methods-based-on-crispr-cas>).

An alternative method to confirm active infections is by a sandwich enzyme-linked immunosorbent assay (ELISA; Fig. 1a). This assay detects viral protein (*i.e.* antigen) in clinical specimens after lysis of the cells and virus particles in the sample. The resulting solution is applied to a well coated with a so-called capture antibody specific for one of the virion proteins. Next, an antibody recognizing another epitope of the same virion protein (the so-called detection antibody) is applied to the well, followed by an enzyme-conjugated third antibody directed against the detection antibody. A colourless substrate is then added to the well, which can be converted by the enzyme into a coloured product that is detected with a colorimeter. The amount of coloured product that is generated correlates with the amount of virus present in the clinical specimen. Since a sandwich ELISA is a laborious procedure requiring multiple washing steps after each addition, which are usually performed automatically, this method is unsuitable for point-of-care testing. This has led to the development of immunochromatography strips for fast instrument-free testing employing capillary movement (Fig. 1b). These strips contain a sample pad for specimen application followed by a conjugate pad containing an antigen-specific antibody linked to, for example, colloidal gold or latex particles (first antibody). If the sample contains antigens, complexes are formed with the first antibody, which migrate to the so-called test line, where a fraction of these complexes are bound by another antigen-specific antibody covalently attached to the strip (second antibody), resulting in the formation of a coloured line. The remaining complexes and non-complexed first antibodies migrate further until they reach the so-called control line, which contains covalently attached antibodies recognizing the first antibody (third antibody). This results in the formation of a second coloured line, irrespective of the presence of antigen in the clinical specimen.

In general, antigen detection assays are less sensitive than genome detection methods, resulting in a higher rate of false-negative results. Moreover, whereas RT-qPCR tests and sandwich ELISAs can provide a reasonable estimate of virus load, immunochromatography tests do not allow accurate quantitative analyses. In early infections the amount of SARS-CoV-2 in specimens from the upper part of the respiratory tract may be too low to be detected and repeated sampling may be necessary.

Virological tests are not only instrumental into determining whether somebody is infected with SARS-CoV-2 but also can be used for monitoring disease progression (by taking samples at different sites), to identify virus shedders and for taking deisolation decisions.

**
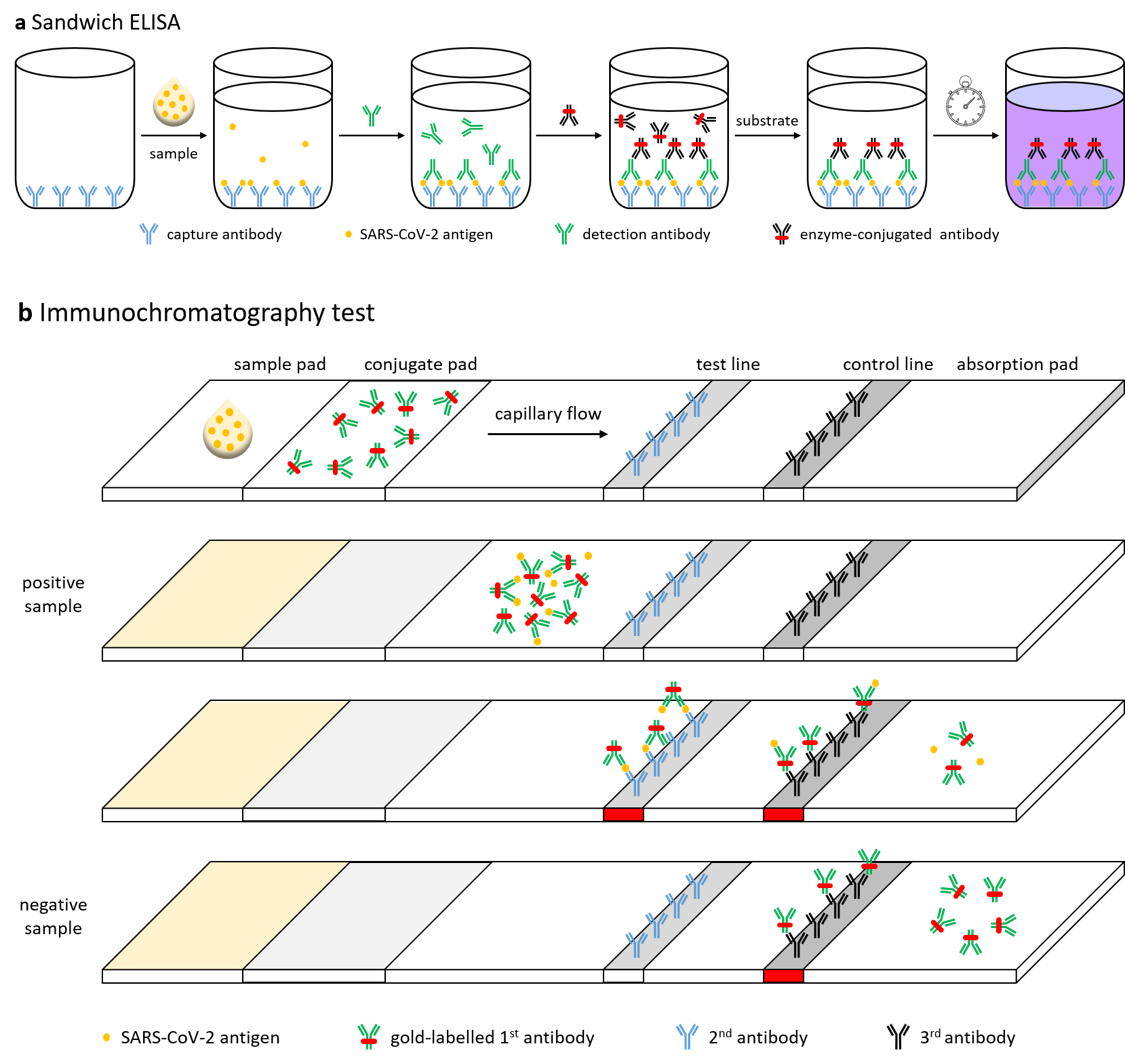
**

**Fig. 1** Antigen detection methods. (A) Sandwich ELISA. (B) Immunochromatography test

*Serological tests*

Different studies report somewhat different times after the onset of symptoms at which the first antibodies against SARS-CoV-2 proteins are detected. Moreover, the extent and kinetics of the humoral immune response to SARS-CoV-2 varies between individuals. This may relate to differences in infectious dose but also be the result of inherited and acquired differences in their immune system. In most studies, IgM, IgG and IgA antibodies directed against the SARS-CoV-2 S and N proteins are first detected at roughly the same time, *i.e.* a few days after the onset of symptoms. IgM and IgG levels peak at 2-3 weeks after illness onset and drop faster afterwards for IgMs (see also above). Serological tests alone can thus not be used to establish with certainty that a person is infected with SARS-CoV-2. The formats of antibody detection tests are similar to those of the antigen detection assays except that viral proteins (most S or N, but sometimes also whole virus lysates) serve as bait. Using whole virus lysates for capturing antibodies may increase the sensitivity of the assay but at the same time reduce its specificity *e.g.* by binding cross-reacting antibodies induced by other human CoVs [18].

Serological tests are particularly useful in surveillance and containment programmes and for epidemic forecasting. They can also be used for assessment of immunity resulting from natural infection or active immunization. However, it is not yet clear to what extent Ig levels correlate with protective immunity. In this respect, virus neutralization assays (*e.g.* plaque reduction and tissue culture infectious dose 50 assays) may be more informative. In these cell culture assays, virus is mixed with (different dilutions) of patient’s (or control) serum and added to susceptible cells to determine whether the serum contains antibodies that can inhibit virus infection.

A comprehensive overview of (1) diagnostic tests, (2) performance data of commercially available diagnostic assays, including information on their sensitivity and specificity and (3) use case descriptions of diagnostic tests for COVID-19 can be found at <https://www.finddx.org/covid-19/>.

**References**

1. Li Q, Guan X, Wu P, et al. Early transmission dynamics in Wuhan, China, of novel coronavirus-infected pneumonia. N Engl J Med. 2020;382:1199‐207.
2. Endo A, Centre for the Mathematical Modelling of Infectious Diseases COVID-19 Working Group, Abbott S et al. Estimating the overdispersion in COVID-19 transmission using outbreak sizes outside China. Wellcome Open Res. 2020;5:67. doi: https://doi.org/10.12688/wellcomeopenres.15842.2. Latest published July 3, 2020.
3. Prather KA, Wang CC, Schooley RT. Reducing transmission of SARS-CoV-2. Science. 2020; 368:1422-4.
4. He X, Lau EHY, Wu P, et al. Temporal dynamics in viral shedding and transmissibility of COVID-19. Nat Med. 2020;26:672‐5.
5. Liu Y, Yan LM, Wan L, et al. Viral dynamics in mild and severe cases of COVID-19. Lancet Infect Dis. 2020;20:656‐7.
6. Chin AWH, Chu JTS, Perera MRA, et al. Stability of SARS-CoV-2 in different environmental conditions. The Lancet Microbe. 220:1:E10.
7. Amirian ES. Potential fecal transmission of SARS-CoV-2: current evidence and implications for public health. Int J Infect Dis. 2020;95:363‐70.
8. Ding S, Liang TJ. Is SARS-CoV-2 also an enteric pathogen with potential fecal-oral transmission: a COVID-19 virological and clinical review. Gastroenterology. 2020;S0016-5085(20)30571-0. doi:10.1053/j.gastro.2020.04.052.
9. Lamers MM, Beumer J, van der Vaart J, et al. SARS-CoV-2 productively infects human gut enterocytes [published online ahead of print, 2020 May 1]. Science. 2020;eabc1669. doi:10.1126/science.abc1669.
10. Xiao F, Tang M, Zheng X, et al. Evidence for gastrointestinal infection of SARS-CoV-2. Gastroenterology. 2020;158:1831‐3.
11. Yang Z, Liu Y. Vertical transmission of severe acute respiratory syndrome coronavirus 2: a systematic review [published online ahead of print, 2020 May 13]. Am J Perinatol. 2020;10.1055/s-0040-1712161. doi:10.1055/s-0040-1712161.
12. Shi J, Wen Z, Zhong G, et al. Susceptibility of ferrets, cats, dogs, and other domesticated animals to SARS-coronavirus 2. Science. 2020;368:1016‐20.
13. Merad M, Martin JC. Pathological inflammation in patients with COVID-19: a key role for monocytes and macrophages. Nat Rev Immunol. 2020;20:355‐62.
14. Netea MG, Giamarellos-Bourboulis EJ, Domínguez-Andrés J, et al. Trained immunity: a tool for reducing susceptibility to and the severity of SARS-CoV-2 infection. Cell. 2020;181:969‐77.
15. Risitano AM, Mastellos DC, Huber-Lang M, et al. Complement as a target in COVID-19? Nat Rev Immunol. 2020;20:343‐4.
16. Tay MZ, Poh CM, Rénia L, et al. The trinity of COVID-19: immunity, inflammation and intervention. Nat Rev Immunol. 2020;20:363-74.
17. Cao X. COVID-19: immunopathology and its implications for therapy. Nat Rev Immunol. 2020;20:269‐70.
18. Long QX, Liu BZ, Deng HJ, et al. Antibody responses to SARS-CoV-2 in patients with COVID-19. Nat Med. 2020; 26:845-8.
19. Padoan A, Sciacovelli L, Basso D, et al. IgA-Ab response to spike glycoprotein of SARS-CoV-2 in patients with COVID-19: a longitudinal study. Clin Chim Acta. 2020;507:164‐6.
20. Grifoni A, Weiskopf D, Ramirez SI, et al. Targets of T cell responses to SARS-CoV-2 coronavirus in humans with COVID-19 disease and unexposed individuals. Cell. 2020;181:1489-501.
21. Ni L, Ye F, Cheng ML, et al. Detection of SARS-CoV-2-specific humoral and cellular immunity in COVID-19 convalescent individuals. Immunity. 2020;52:971-7.
22. Cheng MP, Papenburg J, Desjardins M, et al. Diagnostic testing for severe acute respiratory syndrome-related coronavirus 2: a narrative review. Ann Intern Med. 2020;172:726‐34.
23. Venter M, Richter K. Towards effective diagnostic assays for COVID-19: a review. J Clin Pathol. 2020;73:370-7.
